# Methodology of Natsal-COVID Wave 2: A large, quasi-representative, longitudinal survey measuring the impact of COVID-19 on sexual and reproductive health in Britain

**DOI:** 10.12688/wellcomeopenres.17850.2

**Published:** 2024-01-08

**Authors:** Emily Dema, Anne Conolly, Malachi Willis, Andrew J. Copas, Soazig Clifton, Margaret Blake, Julie Riddell, Raquel Bosó Pérez, Clare Tanton, Chris Bonell, Pam Sonnenberg, Catherine H. Mercer, Kirstin R. Mitchell, Nigel Field

**Affiliations:** 1 Institute for Global Health, University College London, Mortimer Market Centre, London, WC1E 6JB, UK; 2 NatCen Social Research, 35 Northampton Square, London, EC1V 0AX, UK; 3 MRC/CSO Social and Public Health Sciences Unit, University of Glasgow, 99 Berkeley Street, Glasgow, G3 7HR, UK; 4 Ipsos, 3 Thomas More Square, London, E1W 1YW, UK; 5 Faculty of Public Health and Policy, London School of Hygiene and Tropical Medicine, Keppel Street, London, WC1E 7HT, UK

**Keywords:** COVID-19, population estimates, online survey, cross-sectional and longitudinal data, sexual behaviour, sexual health, relationships

## Abstract

**Background:**

The National Surveys of Sexual Attitudes and Lifestyles COVID study (Natsal-COVID) was designed to understand the impact of COVID-19 on Britain’s sexual and reproductive health (SRH). Natsal-COVID Wave 1 survey and qualitative follow-up interviews were conducted in 2020. The Wave 2 survey was designed to capture one-year prevalence estimates for key SRH outcomes and measure changes over the first year of the pandemic. We describe the Wave 2 survey methodology and assess the sample representativeness.

**Methods:**

Natsal-COVID Wave 2 was conducted March-April 2021; approximately one year after the start of Britain’s first national lockdown. Data were collected using an online web-panel survey administered by Ipsos. The sample comprised a longitudinal sample of Wave 1 participants who had agreed to re-contact plus a sample of participants residing in Britain, aged 18-59, including a boost sample comprising people aged 18-29. Questions covered reproductive health, relationships, sexual behaviour and SRH service use. Quotas and weighting were used to achieve a quasi-representative sample of the British population. Comparisons were made with recent national probability surveys, Natsal-3 (2010-12) and Natsal-COVID Wave 1 to understand bias.

**Results:**

A total of 6,658 individuals completed the survey. In terms of gender, age, ethnicity, and rurality, the weighted Natsal-COVID Wave 2 sample was like the general population. Participants were less likely to be married or to report being in good health than the general population. The longitudinal sample (n=2,098) were broadly like participants who only took part in Wave 1 but were older. Among the sexually active, longitudinal participants were less likely to report multiple sexual partners or a new sexual partner in the past year compared to those who only took part in Wave 1.

**Conclusions:**

Natsal-COVID collected longitudinal, quasi-representative population data to enable evaluation of the population-level impact of COVID-19 on SRH and to inform policy.

## Background

This research note describes the methodology used to conduct Wave 2 of the National Surveys of Sexual Attitudes and Lifestyles COVID study (Natsal-COVID). It follows our previous work describing the Wave 1 study methodology ^1^.

At the time of Natsal-COVID Wave 2 data collection (March–April 2021), COVID-19 lockdown restrictions in Britain were being eased following a second national lockdown between January and March 2021. Some form of restrictions and physical distancing requirements had been in place throughout the preceding year ^2^. Physical contact with anyone outside one’s household or support bubble was not permitted for the duration of that year.

Natsal-COVID survey Wave 1 and qualitative follow-up interviews were conducted in 2020 ^1^ to understand early changes in sexual and reproductive health (SRH) service use and need, sexual behaviours, and relationships during this time ^3– 6^. Wave 2 aimed to capture SRH behaviour and outcomes during the first year of the COVID-19 pandemic, including sexual behaviours, sexual function, relationship quality, intimate partner violence, reproductive health outcomes and SRH service use. It was designed to produce one-year prevalence estimates of key SRH outcomes and behaviours. The study also aimed to measure change over time, both between-person variation through repeat cross-sectional analyses and within-person variation through longitudinal analyses. This paper describes the methods used in Wave 2 of Natsal-COVID and assesses the representativeness of the data.

## Sample design

Natsal-COVID Wave 1 was an online web-panel survey conducted in July–August 2020, which used quotas and weighting to obtain a quasi-representative sample of 6654 people aged 18–59 years old living in Britain. The Wave 2 sample was drawn first from those who participated in Wave 1 and agreed to re-contact (the longitudinal sample). No quotas were set for this group. To complete the Wave 2 sample, new participants were sampled from Ipsos’s online panels. Sample quotas were set by gender, age, region, and social grade. The new sample included a boost of 500 people aged 18–29 years old, ensuring an overall sample of 2000 participants in this age-group. The complete Wave 2 sample was designed to ensure overall representativeness of the population aged 18–59 years old by age, gender, region, and social grade. Full details of sample size calculations for Natsal-COVID have been reported elsewhere ^1^. The target longitudinal sample size at Wave 2 was 4,000.

## Ethical approval

We obtained ethics approval for the study from University of Glasgow Medical, Veterinary and Life Sciences College Ethics Committee (reference 20019174) and London School of Hygiene and Tropical Medicine Research Ethics committee (reference 22565). Participants provided informed consent to participate via an online consent form before starting the survey.

## The questionnaire

The Natsal-COVID Wave 2 questionnaire was adapted from the Wave 1 questionnaire, the development of which has been previously described ^1^. The dataset for Wave 1 can be found in the UK Data Archive ^7^. Wave 2 additionally included questions about pregnancy, contraception changes, HIV testing, chlamydia testing, abortion, relationship formation and dissolution, and IPV with a focus on the year since the start of the first lockdown ( Box 1). Questions relating to experiences of the COVID-19 pandemic were included, mainly drawn from other major COVID-19 studies ^8, 9^. The full questionnaire is available on the study website under ‘ Natsal-COVID Survey Questionnaire - Wave 2’.

**Box 1. National Surveys of Sexual Attitudes and Lifestyles COVID study (Natsal-COVID) Wave 2 questionnaire content**

Gender identity, sex at birth

Who you’ve been living with since lockdown

General health and disability

COVID-19: testing positive, vaccination, pandemic impact

Alcohol consumption

Mental health - Generalised anxiety disorder two item (GAD-2) and patient health questionnaire two item (PHQ-2) scales, loneliness

Ethnicity, country of birth

Sexual identity

Employment status

Education

Number of opposite-sex, same-sex, and transgender partners in different time periods (past 5 years, since lockdown)

Condomless sex with new opposite-sex, same-sex, and transgender partners since lockdown

Sexual behaviours since lockdown

Sexual function

Pregnancy (including the London Measure of Unplanned Pregnancy)

Chlamydia testing

Access to sexual and reproductive health (SRH) services

Unmet need for SRH services

Method of accessing sexually transmitted infection (STI) testing services

Contraception used since lockdown

Condom access since lockdown

Changes in sexual relationships since lockdown

Intimate relationships and difficulties

Intimate Partner Violence

Natsal surveys have always involved sensitive question topics, *e.g.*, sexual behaviours, reproductive health, SRH service use. The introduction of IPV questions using remote collection methods in Natsal-COVID Wave 2 required careful consideration regarding potential risk to participants and data quality, while acknowledging the importance of collecting these data during the COVID-19 pandemic. A variety of measures were put in place to mitigate the risks. We minimised the number of IPV items, avoided very sensitive questions ( *e.g.*, physical force) and included an explicit ‘prefer not to answer’ option at every question in the module. Immediately before sensitive question modules (including IPV), reminders about the voluntary nature of each question and confidentiality were displayed. Appropriate signposting to support services was provided after each sensitive module and at the end of the survey. 

## Sample recruitment

**Figure 1.**
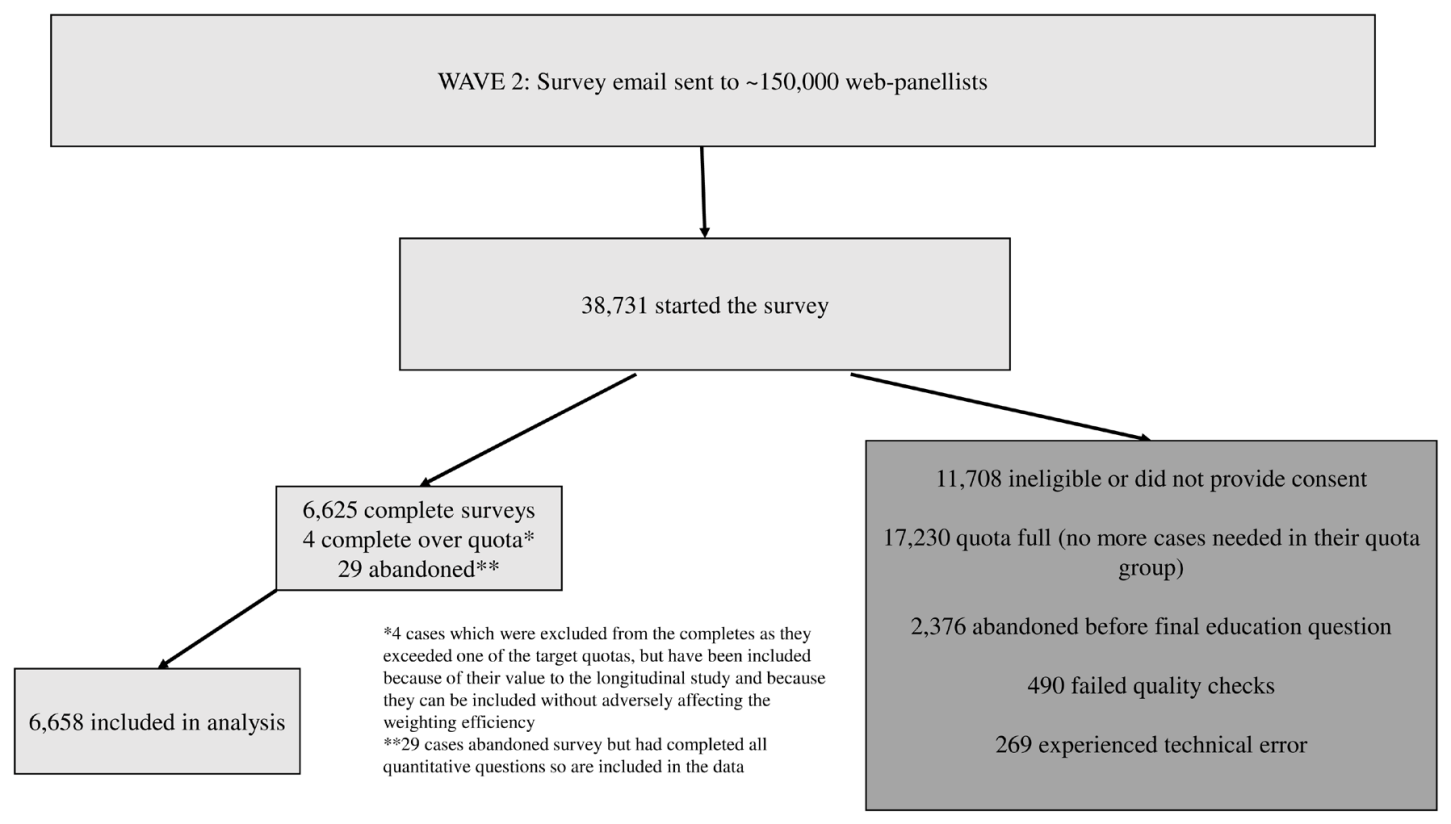
Recruitment process for National Surveys of Sexual Attitudes and Lifestyles COVID study (Natsal-COVID) Wave 2.

Wave 2 survey data were collected from 27 March 2021 to 26 April 2021. Approximately 150,000 panellists, including those from Wave 1 who were willing to be re-contacted, were contacted via email. Of those emailed, 38,731 started the survey; 79% came from Ipsos’s own panel, with ‘top-up’ from three other panel providers used by Ipsos. Ipsos reports never depending solely on any one method or source of recruitment, and they reach different types of people through different methodologies and suppliers. Methods include partnering with affiliate networks which run recruitment campaigns across 40–50 of their own member websites at any given time. Performance measures are used to adjust recruitment sources and they discontinue partnering with sources that provide poor-quality respondents. Of these participants, 11,708 were ineligible or did not provide consent, 17,230 were diverted from completing the survey because their quota was full, 2,376 abandoned the survey before completion, 490 failed quality checks, and 269 experienced a technical error. In total, 6,658 participants completed the survey and are included in the analysis. Of these, 2,098 were longitudinal participants and 4,556 were new. The Ipsos online panels are run with stringent recruitment processes and quality control to ensure individuals can only take part once, are not oversampled and are engaged. Checks are used at recruitment and while people are on the panel to ensure bad and inactive panellists are removed. Cases which display fraudulent or straight lining behaviour during the survey are removed from the data. The recruitment process is shown in Figure 1.

Participants completed the survey on a smartphone (53%), a laptop/desktop (42%) or a tablet (6%).

## Quota filling and weighting of survey data

To increase numbers of participants aged 18–29 years old, all other quotas (gender, region, and social grade) were relaxed toward the end of fieldwork. Cross-sectional and longitudinal weights were produced to achieve a quasi-representative sample of the British population by gender, age, region, social grade, ethnicity, and sexual identity. The cross-sectional weight for the full Wave 2 sample had a weighting efficiency of 83.8% (see Ipsos Wave 2 Technical Report).

## Gender in Natsal-COVID

**Figure 2.**
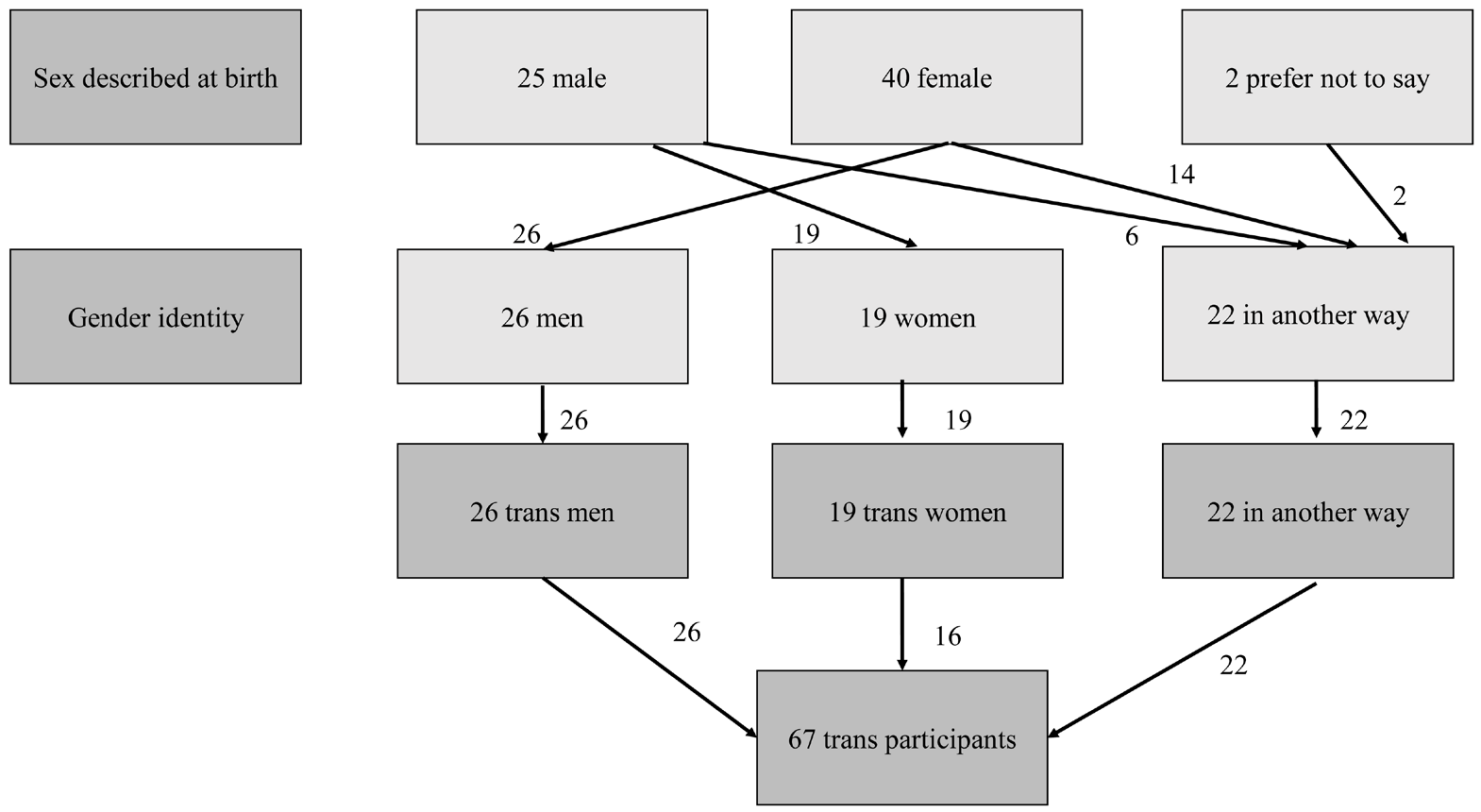
Classification of trans participants in National Surveys of Sexual Attitudes and Lifestyles COVID study (Natsal-COVID) Wave 2.

Natsal-COVID is inclusive in its approach to gender, as described for Wave 1 ^1^. A total of 67 Wave 2 participants were classified as ‘trans’ where their reported gender identity was different to their sex described at birth. This included 26 trans men, 19 trans women, and 22 people who identified in another way ( Figure 2), giving an overall percentage of 0.9% in the weighted sample. We present data for men (including trans men) and women (including trans women) in our analysis. Individuals who identified ‘in another way’ were included in analyses where the denominator is all participants but were not included in denominators for men or women.

## Representativeness of the Natsal-COVID sample

The Natsal-COVID Wave 2 sample was compared with Wave 1, and with the following probability sample surveys to assess representativeness ( Table 1 and Table 2): the 2019 Annual Population Survey (APS) ^10^ (gender, age, region, ethnicity, marital status, education, disability), the 2018 Health Survey for England (HSE) ^11^ (general health and rurality), the 2018 APS report ^12^ (sexual identity), and the 2010-12 Natsal-3 study ^13^ (sexual behaviours). More recent versions of HSE and APS have been published since we carried out analysis of Natsal-COVID Wave 1 representativeness. However, we decided to use consistent comparators in our assessment of the Natsal-COVID Wave 2 sample to facilitate comparisons with Wave 1. The archived datasets were accessed from the UK Data Archive and Office for National Statistics (ONS) website. Data analysts (ED, SC, JR) had access to the datasets.

**Table 1.** National Surveys of Sexual Attitudes and Lifestyles COVID study (Natsal-COVID) Wave 1 and Wave 2 unweighted and weighted distributions of quota and weighting variables compared with external probability surveys.

		Men (including Trans Men)					Women (including Trans Women)					All				
		% [95% CI]					% [95% CI]					% [95% CI]				
		Natsal-COVID Wave 2		Natsal-COVID Wave 1		Population estimate ^1^	Natsal-COVID Wave 2		Natsal-COVID Wave 1		Population estimate ^1^	Natsal-COVID Wave 2		Natsal-COVID Wave 1		Population estimate ^1^
**Denominators (weighted, ** **unweighted)**		3312, 3095		3310, 3187		17520655, 61993	3322, 3541		3320, 3443		17668414, 68988	6658, 6658		6654, 6654		35189069, 130981
		*Unweighted %*	*Weighted % * *[95% CI]*	*Unweighted %*	*Weighted % * *[95% CI]*		*Unweighted %*	*Weighted % * *[95% CI]*	*Unweighted %*	*Weighted % * *[95% CI]*		*Unweighted %*	*Weighted % * *[95% CI]*	*Unweighted %*	*Weighted % * *[95% CI]*	
**Gender** ^2^	Men	-	-	-	-	-	-	-	-	-	-	46.5	49.8 [48.4, 51.1]	47.9	49.8 [48.5,51.0]	49.8 [49.5,50.1]
	Women	-	-	-	-	-	-	-	-	-	-	53.2	49.9 [48.6, 51.2]	51.7	49.9 [48.6,51.2]	50.2 [49.9,50.5]
	Trans	0.8	0.6 [0.4, 0.9]	0.7	0.6 [0.4,0.9]	-	0.5	0.5 [0.3, 0.9]	0.4	0.4 [0.2, 0.7]	-	1.0	0.9 [0.7, 1.2]	0.9	0.8 [0.6,1.1]	-
**Age**	18–24	15.5	16.1 [14.7, 17.7]	12.6	13.6 [12.3,15.0]	15.6 [15.3,16.0]	15.1	12.7 [11.6, 13.9]	17.3	12.0 [11.1,13.1]	14.8 [14.5,15.2]	15.3	14.5 [13.6, 15.5]	15.2	12.9 [12.1,13.8]	15.2 [15.0,15.5]
	25–34	21.1	21.6 [20.0, 23.2]	24.3	25.5 [23.91,27.2]	25.0 [24.6,25.4]	29.9	26.2 [24.8, 27.7]	33.3	27.6 [26.1,29.2]	24.6 [24.2,25.0]	25.8	23.9 [22.9, 25.0]	29	26.6 [25.5,27.7]	24.8 [24.5,25.1]
	35–44	24.0	25.8 [24.1, 27.5]	23.1	23.8 [22.3,25.5]	22.9 [22.5,23.3]	23.6	23.5 [22.1, 25.0]	21.4	24.5 [23.0,26.1]	23.2 [22.8,23.6]	23.8	24.6 [23.5, 25.8]	22.2	24.1 [23.0,25.3]	23.1 [22.8,23.3]
	45–59	39.4	36.5 [34.7, 38.4]	40.0	37.1 [35.3,38.8]	36.5 [36.0,36.9]	31.5	37.5 [35.8, 39.3]	28	35.89 [34.1,37.7]	37.4 [27.0,37.8]	35.1	36.9 [35.7, 38.2]	33.7	36.4 [35.1,37.6]	36.9 [36.6,37.2]
	Median (IQR) [95 ^th^ percentile]	39 (28, 51) [58]	39 (28, 49) [58]	40 (29, 51) [58]	39 (29, 49) [58]	38 (28, 49) [57]	36 (28, 47) [58]	39 (29, 50) [58]	34 (26, 46) [58]	39 (29, 50) [58]	39 (29, 50) [57]	38 (28, 49) [58]	39, (28, 49) [58]	37 (28, 48) [58]	39 (29, 49) [58]	39 (29, 49) [57]
**Region**	England	87.6	86.8 [85.4, 88.1]	89	86.9 [85.4,88.2]	86.9 [86.7,87.2]	87.4	86.6 [85.3, 87.8]	88	86.6 [85.3,87.9]	86.6 [86.4,86.9]	87.5	86.7 [85.8, 87.6]	88.5	86.7 [85.8,87.6]	86.8 [86.6,87.0]
	Wales	4.4	4.7 [3.9, 5.6]	3.9	4.7 [3.9,5.6]	4.7 [4.6,4.8]	4.5	4.7 [4.0, 5.5]	3.9	4.7 [3.9,5.6]	4.7 [4.6,4.8]	4.5	4.7 [4.2, 5.3]	3.9	4.7 [4.1,5.3]	4.7 [4.6,4.8]
	Scotland	8	8.5 [7.4, 9.6]	7.2	8.5 [7.4,9.7]	8.4 [8.1,8.6]	8.1	8.7 [7.7, 9.8]	8.1	8.7 [7.7,9.8]	8.7 [8.5,8.9]	8.1	8.6 [7.9, 9.4]	7.7	8.6 [7.9,9.4]	8.5 [8.4,8.7]
**Social ** **grade ^2^**	A Upper middle class/ B Middle class	34.8	23.1 [21.7, 24.5]	25.6	23.1 [21.6,24.6]	-	23.9	22.2 [20.8, 23.7]	24.2	22.2 [20.8,23.7]	-	29	22.7 [21.7, 23.7]	24.8	22.6 [21.6,23.7]	-
	C1 Lower middle class/C2 Skilled working class	41.1	53.0 [51.1, 54.9]	52.6	53.0 [51.2,54.9]	-	49.6	52.4 [50.6, 54.1]	50.9	52.4 [50.6,54.2]	-	45.7	52.7 [51.4, 54.0]	51.7	52.7 [51.4,54.0]	-
	D Working class/E Lower level of subsistence	24.1	23.9 [22.3, 25.6]	21.8	23.9 [22.3,25.6]	-	26.4	25.4 [23.9, 26.9]	25	25.4 [23.9,27.0]	-	25.3	24.7 [23.6, 25.8]	23.4	24.7 [23.5,25.8]	-
**Ethnicity**	White ^3^	86.9	85.6 [84.0, 87.0]	89.5	85.6 [84.0,87.1]	84.7 [84.4,85.1]	88	85.7 [84.4, 87.1]	89.4	85.8 [84.3,87.2]	83.8 [83.4,84.1]	87.5	85.7 [84.7, 86.7]	89.4	85.7 [84.6,86.7]	84.3 [84.0,84.5]
	Mixed/multiple ^4^	2.4	1.7 [1.3, 2.1]	1.9	1.7 [1.3,2.2]	1.3 [1.2,1.4]	2.6	1.7 [1.4, 2.1]	2.3	1.7 [1.3,2.2]	1.4 [1.3,1.5]	2.5	1.7 [1.4, 2.0]	2.1	1.7 [1.4,2.0]	1.3 [1.3,1.4]
	Asian/Asian British ^5^	7.2	8.2 [7.2, 9.4]	6.4	8.2 [7.2,9.4]	8.4 [8.2,8.7]	6.3	8.0 [7.0, 9.1]	5.7	8.0 [6.9,9.2]	8.6 [8.4,8.9]	6.7	8.1 [7.3, 8.9]	6.1	8.11 [7.4,8.9]	8.5 [8.3,8.7]
	Black/Black British ^6^	3.1	3.3 [2.6, 4.0]	1.8	3.3 [2.5,4.2]	3.4 [3.3,3.6]	2.5	3.5 [2.8, 4.4]	2.1	3.5 [2.8,4.5]	4.0 [3.8,4.2]	2.8	3.4 [2.9, 4.0]	2	3.4 [2.8,4.0]	3.7 [3.6,3.9]
	Other	0.4	1.3 [0.7, 2.3]	0.4	1.3 [0.7,2.2]	2.1 [2.0,2.3]	0.5	0.9 [0.6, 1.5]	0.5	0.9 [0.6,1.5]	2.2 [2.0,2.3]	0.5	1.1 [0.7, 1.7]	0.5	1.1 [0.8,1.6]	2.1 [2.0,2.2]
**Sexual ** **identity**	Heterosexual/ straight	88	96.2 [95.7, 96.7]	86.8	96.2 [95.7,96.6]	94.4 [94.1, 94.7]	89.2	96.4 [95.9, 96.8]	89.5	96.4 [95.9,96.8]	94.9 [94.7,95.1]	88.5	96.0 [95.6, 96.3]	87.9	96.0 [95.6, 96.3]	94.6 [94.4, 94.8]
	Gay/Lesbian	6.8	2.3 [2.0, 2.7]	7.9	2.4 [2.1,2.7]	1.9 [1.7,2.1]	2.6	1.1 [0.8, 1.4]	2.2	1.1 [0.8,1.4]	0.9 [0.8,1.0]	4.6	1.8 [1.6, 2.0]	5	1.8 [1.4,2.0]	1.4 [1.3, 1.5]
	Bisexual	4.2	0.9 [0.7, 1.1]	4.6	0.9 [0.8,1.1]	0.6 [0.5,0.7]	6.7	1.8 [1.5, 2.0]	7.2	1.8 [1.5,2.0]	1.1 [1.0,1.2]	5.6	1.4 [1.3, 1.6]	6	1.4 [1.3,1.6]	0.9 [0.8, 1.0]
	Other	0.9	0.6 [0.3, 1.0]	0.8	0.6 [0.4,0.9]	0.5 [0.4,0.6]	1.5	0.8 [0.6, 1.1]	1.3	0.8 [0.5,1.1]	0.6 [0.5,0.7]	1.4	0.8 [0.6, 1.1]	1.1	0.8 [0.6,1.0]	0.6 [0.5, 0.7]

Legend: CI=confidence intervals. 1. Population estimate comparisons use the 2019 Annual Population Survey (APS). Analysis was restricted to those aged 18–59 resident in Britain. This was not possible for ‘sexual identity’ where microdata were unavailable and estimates were taken from aggregated data of individuals aged 16+ in the UK. 2. Here, trans men and trans women are included in the trans row; men and women rows refer to those whose gender identity is the same as their sex described at birth. No external comparison data available for the trans population. 3. White includes all those who identify as White English, Welsh, Scottish, Northern Irish, British, Irish, Gypsy or Irish Traveller, or from any other White background. 4. Mixed ethnicity includes those who identify as White and Black African, White and Black Caribbean, White and Asian, or any other mixed or multiple ethnic background. 5. Asian includes those who identify as Indian, Pakistani, Bangladeshi, Chinese or from any other Asian background. 6. Black includes those who identify as African, Caribbean, or from any other Black background.

**Table 2.** National Surveys of Sexual Attitudes and Lifestyles COVID study (Natsal-COVID) Wave 1 and Wave 2 distributions compared with external probability surveys and Natsal-3 data.

		Men (including Trans Men)					Women (including Trans Women)					All				
		% [95% CI]					% [95% CI]					% [95% CI]				
		Natsal-COVID Wave 2		Natsal-COVID Wave 1		Population estimate	Natsal-COVID Wave 2		Natsal-COVID Wave 1		Population estimate	Natsal-COVID Wave 2		Natsal-COVID Wave 1		Population estimate
**Denominators (weighted, ** **unweighted)**		3312, 3095		3310, 3187		17520655, 61993	3322, 3541		3320, 3443		17668414, 68988	6658, 6658		6654, 6654		35189069, 130981
		*Unweighted %*	*Weighted % * *[95% CI]*	*Unweighted %*	*Weighted % * *[95% CI]*		*Unweighted %*	*Weighted % * *[95% CI]*	*Unweighted %*	*Weighted % * *[95% CI]*		*Unweighted %*	*Weighted % * *[95% CI]*	*Unweighted %*	*Weighted % * *[95% CI]*	
**Married ^1^**	Yes	40.6	40.3 [38.4, 42.3]	38.7	39.8 [37.9,41.6]	46.8 [46.4,47.3]	37.3	40.7 [38.9, 42.5]	36	41.4 [39.6,43.2]	48.2 [47.7,48.6]	38.7	40.4 [39.1, 41.7]	37.2	40.5 [39.2,41.7]	47.5 [47.2,47.8]
**Education ^2, 3^**	No qualification	5.4	5.7 [4.8, 6.8]	4.1	4.3 [3.5,5.1]	11.7 [10.3,13.2]	4.4	4.5 [3.8, 5.4]	3.2	3.5 [2.8,4.3]	11 [9.7,12.3]	4.8	5.1 [4.5, 5.8]	3.6	3.9 [3.4,4.4]	11.3 [10.4,12.3]
	Below degree	48.3	51.7 [49.6, 53.8]	50	49.9 [48.0,51.9]	54.8 [52.5,57.1]	49.8	50.1 [482, 51.9]	46.6	47.1 [45.2,49.0]	53 [51.0,54.9]	49.1	50.9 [49.5, 52.3]	48.3	48.6 [47.2,49.9]	34.8 [33.4,36.3]
	Degree	46.3	42.6 [40.5, 44.7]	46	45.9 [43.9,47.8]	33.5 [31.4,35.7]	45.8	45.4 [43.6, 47.3]	50.2	49.4 [47.5,51.4]	36.1 [34.2,38.0]	46	44.0 [42.6, 45.4]	48.1	47.6 [46.2,48.9]	53.9 [52.4,55.4]
**Rurality ^2^**	Urban	87.5	87.1 [85.5, 88.5]	87	87.4 [86.0,88.7]	84.3 [82.7,85.7]	84.8	85.2 [83.7, 86.6]	84.8	85.1 [83.6,86.5]	84 [82.6,85.3]	86	86.1 [85.1, 87.1]	85.9	86.3 [85.3,87.3]	84.1 [83.1,85.1]
	Rural	12.5	12.9 [11.5, 14.5]	13	12.6 [11.3,14.0]	15.7 [14.3,17.3]	15.2	14.8 [13.4, 16.3]	15.2	14.9 [13.5,16.4]	16.0 [14.7,17.4]	14	13.9 [12.9, 14.9]	14.1	13.7 [12.7, 14.7]	15.9 [14.9,16.9]
**General health ** **status ^2^**	Good/ Very Good	70.4	70.1 [68.2, 72.0]	73.1	74.0 [72.3,75.7]	81.3 [79.5,82.9]	70	70.5 [68.7, 72.2]	73.3	72.9 [71.1,74.5]	78.6 [77.0,80.1]	70.1	70.2 [68.9, 71.5]	73.1	73.3 [72.1,74.5]	79.9 [78.7,81.1]
	Fair	23.4	23.8 [22.0, 25.6]	21	20.4 [18.9,22.0]	13 [11.7,14.5]	23.1	22.7 [21.1, 24.3]	21.6	21.8 [20.3,23.4]	15.2 [13.8,16.6]	23.3	23.3 [22.1, 24.5]	21.3	21.1 [20.0,22.3]	14.1 [13.1,15.1]
	Bad/ Very bad	6.2	6.1 [5.2, 7.2]	5.9	5.6 [4.8,6.5]	5.7 [4.7,6.9]	6.9	6.8 [5.9, 7.9]	5.2	5.3 [4.5,6.3]	6.2 [5.4,7.2]	6.6	6.5 [5.8, 7.2]	5.6	5.6 [5.0,6.2]	6.0 [5.3,6.7]
**Limiting long-** **term illness/** **disability ^1^**	Yes	31	29.9 [28.1, 31.8]	31.1	28.9 [27.2,30.6]	28.4 [28.0,28.8]	37.6	36.2 [34.5, 37.9]	37	35.8 [34.1,37.6]	32.5 [32.1,32.9]	34.7	33.2 [32.0, 34.5]	34.2	32.5 [31.3,33.7]	30.5 [30.2,30.8]
**Ever had any ** **partnered ** **sexual ** **experience ** **(not ** **necessarily ** **involving ** **genital ** **contact) ^4, 5^**	Yes	86.9	86.3 [84.9, 87.6]	91	90.5 [89.3,91.6]	-	88.6	88.2 [87.0, 89.3]	90	89.4 [88.1,90.5]	-	87.8	87.2 [86.3, 88.1]	90.4	89.9 [89,90.6]	-
**Ever had any ** **partnered ** **sexual ** **experience ** **(modified for ** **comparison ** **with N-3) ^4, 5, 6^**	Yes	94.1	93.8 [92.7, 94.7]	-	-	98.7 [98.4,99.0]	94.2	94.2 [93.2, 94.9]	-	-	98.8 [98.4,99.1]	94.1	93.9 [93.3, 94.5]	-	-	98.8 [98.5,99.9]
**Ever had ** **vaginal, anal, ** **oral sex ** **or other genital ** **contact with a ** **partner of any ** **gender ^4, 5^**	Yes	92.5	92.3 [91.2, 93.3]	85.9	85.1 [83.5, 86.5]	96.6 [96.0,97.0]	92.2	92.3 [91.2, 93.2]	83.1	82.7 [81.2, 84.2]	97.1 [96.6,97.5]	92.3	92.2 [91.5, 92.9]	84.4	83.8 [82.7, 84.9]	96.8 [96.5,97.2]
**Ever had ** **vaginal, anal, ** **oral sex or ** **other genital ** **contact with a ** **same sex ** **partner ^4, 5^**	Yes	11.8	6.4 [5.5, 7.3]	13.2	6.3 [5.6,7.2]	5.7 [5.0,6.5]	9.2	5.8 [5.1, 6.6]	9.1	5.4 [4.7,6.1]	7.3 [6.7,8.0]	10.4	6.1 [5.5, 6.7]	11	5.8 [5.3,6.4]	6.5 [6.0, 7.1]
**Partner ** **numbers, past ** **5 years ^4, 7^**	0 partners	17.2	17.3 [15.7, 18.9]	-	-	3.0 [2.5, 3.6]	15.6	16.6 [15.2, 18.1]	-	-	4.5 [4.0,5.1]	16.3	16.9 [15.9, 18.0]	-	-	3.8 [3.4, 4.2]
	1 partner	50.8	51.2 [49.1, 53.3]	-	-	55.8 [54.1, 57.4]	59.3	61.4 {59.6, 63.2]	-	-	64.6 [63.2, 65.9]	55.2	56.2 [54.8, 57.6]	-	-	60.2 [59.2, 61.2]
	2–4 partners	18.0	19.1 [17.4, 20.9]	-	-	23.8 [22.5, 25.3]	17.0	15.4 [14.1, 16.7]	-	-	21.0 [20.0, 22.1]	17.5	17.3 [16.2, 18.4]	-	-	22.4 [21.6, 23.3]
	5+ partners	14.0	12.5 [11.1, 13.9]	-	-	17.3 [16.2, 18.6]	8.1	6.6 [5.8, 7.6]	-	-	9.9 [9.1, 10.6]	10.9	9.6 [8.8, 10.5]	-	-	13.6 [12.9, 14.3]
	Median (IQR) [95th percentile]	1 (1, 2) [10]	1 (1, 2) [10]	-	-	1 (1, 3) [13]	1 (1, 2) [6]	1 (1, 1) [5]	-	-	1 (1, 2) [7]	1 (1, 2) [9]	1 (1, 2) [8]	-	-	1 (1, 2) [10]

Legend: CI=confidence intervals. 1. Population estimate comparisons use the 2019 Annual Population Survey (APS). Analysis was restricted to those aged 18–59 resident in Britain. 2. Comparison data from 2018 Health Survey for England 2018 (HSE). Analysis was restricted to those aged 18–59 resident in Britain. 3. Natsal-COVID participants chose an option from the following list: (1) primary school, (2) secondary school (age under 15), (3) GNVQ / GSVQ / GCSE/ SCE standard, (4) NVQ1/NVQ2, (5) NVQ3/ SCE Higher Grade/ Advanced GNVQ/ GCE A/AS or similar, (6) NVQ4 / HNC / HND / Bachelor's degree or similar, and (7) NVQ5 or post-graduate diploma. A 3-category education variable, based on a variable used by 2018 Health Survey for England (HSE), including “no qualification,” “below degree”, and “degree” was derived using the following: No qualification: 1–2; Below degree: 3–5; Degree: 6–7. 4. Comparison data from Natsal-3. Analysis was restricted to those aged 18–59 resident in Britain. 5. All respondents (including sexually inexperienced). 6. Sexual experience questions differed between Natsal-COVID and Natsal-3. Natsal-COVID includes reporting any sexual experience (self-defined) or any vaginal, oral or anal sex, or genital contact with a partner. Natsal-3 includes reporting sexual intercourse with an opposite sex partner or oral, anal or genital contact with a same sex partner. 7. All respondents reporting at least one sexual partner in their lifetime (vaginal, anal, oral sex, or other genital contact).

As expected, due to quotas and weighting, the weighted Wave 2 sample was similar to Wave 1 and external datasets for gender, age, region, ethnicity, and sexual identity ( Table 1). Like Wave 1, the unweighted Wave 2 sample over-represented non-heterosexual identifying participants (men, 11.9%; women, 10.8%). However, the weighted sample (men, 3.8%; women, 3.6%) was comparable to 2018 APS (men, 3.0%; women, 2.6%). Sexual identity was not available in the APS dataset, so we relied on reported tabulated data for a comparable population estimate. We have therefore compared individuals aged 18–59 years old in Natsal-COVID to those aged 16+ ( *i.e.*, no upper age limit) in APS. The over-representation of non-heterosexual identities in the unweighted Natsal-COVID sample can be partially explained by the younger age range of participants.

Regarding other sociodemographic variables, patterns were largely similar between Wave 1 and Wave 2. The Wave 2 sample under-represented participants who reported being married (40.4%) compared to the 2019 APS (47.5%) and under-represented those who reported ‘very good’ or ‘good’ general health (73.3%) compared to the 2018 HSE (79.9%).

The weighted proportion of Wave 2 participants reporting any previous partnered sexual experience (not necessarily involving genital contact) (93.9%) was lower than in Natsal-3 (98.8%). Among participants with at least one sexual partner in their lifetime, there was a higher proportion of Natsal-COVID Wave 2 participants reporting zero partners in the past five years (16.9%) compared with Natsal-3 (3.8%).

To characterise the sample of individuals who participated in both Waves of Natsal-COVID, we compared the unweighted and weighted characteristics and behaviours (reported at Wave 1) of those who did not participate in Wave 2 (n=4,556) with those who did (the longitudinal sample; n=2,098) ( Table 3). For most sociodemographic characteristics, the longitudinal sample was similar to those who participated only in Wave 1. However, participants in the youngest age group (18–24) were under-represented in the longitudinal sample (5.1% unweighted; 9.5% weighted) compared to the wave 1 only sample (19.8% unweighted; 17.2% weighted). Conversely, participants in the oldest age group (45–59) were over-represented in the longitudinal sample (49.5% unweighted; 35.4% weighted) compared to Wave 1 only participants (26.4% unweighted; 29.1% weighted). Among sexually active participants, a smaller proportion of the longitudinal sample reported multiple partners between July 2019 and July 2020 (10.9% unweighted; 11.3% weighted) compared with Wave 1 only participants (20.0% unweighted; 17.7% weighted). The longitudinal sample were also less likely to report any new partners in the same timeframe (19.2% unweighted; 23.8% weighted) than those who only took part in Wave 1 (30.8% unweighted; 28.9% weighted).

**Table 3.** National Surveys of Sexual Attitudes and Lifestyles COVID study (Natsal-COVID) Wave 1 only sample compared with the longitudinal sample (Wave 1 and Wave 2).

		Men (including Trans Men)				Women (including Trans Women)				All			
		% [95% CI]				% [95% CI]				% [95% CI]			
		Wave 1 only		Wave 1 and Wave 2 (longitudinal)		Wave 1 only		Wave 1 and Wave 2 (longitudinal)		Wave 1 only		Wave 1 and Wave 2 (longitudinal)	
**Denominators (weighted, ** unweighted)*		2280, 2149		1035, 1038		2212, 2386		1057, 1057		4512, 4556		2095, 2098	
		*Unweighted %*	*Weighted % * *[95% CI]*	*Unweighted %*	*Weighted % * *[95% CI]*	*Unweighted %*	*Weighted % * *[95% CI]*	*Unweighted %*	*Weighted % * *[95% CI]*	*Unweighted %*	*Weighted % * *[95% CI]*	*Unweighted %*	*Weighted % * *[95% CI]*
**Gender**	Men	-	-	-	-	-	-	-	-	47.2	50.5 [49.0, 52.1]	49.5	49.4 [46.6, 52.2]
	Women	-	-	-	-	-	-	-	-	52.4	49.0 [47.5, 50.6]	50.4	50.4 [47.6, 53.3]
	Trans ^2^	0.9	0.7 [0.4, 1.2]	0.4	0.2 [0.1, 0.6]	0.4	0.4 [0.2, 0.8]	0.5	0.8 [0.3, 2.8]	1.1	1.0 [0.8, 1.4]	0.6	0.7 [0.3, 1.6]
**Age**	18–24	16.7	17.8 [16.1, 19.7]	4.1	10.8 [7.7, 14.9]	22.3	16.2 [14.8, 17.6]	6	8.3 [6.3, 10.7]	19.8	17.2 [16.1, 18.4]	5.1	9.5 [7.6, 11.8]
	25–34	29.3	30.1 [28.0, 32.2]	14.1	27.8 [23.3, 32.7]	36.3	31.1 [29.2, 33.1]	26.6	33.4 [30.0, 36.9]	33	30.6 [29.2, 32.0]	20.4	30.7 [27.8, 33.6]
	35–44	22.1	22.8 [20.9, 24.8]	25.1	25.3 [21.9, 28.9]	19.7	23.6 [21.8, 25.6]	25	23.7 [20.9, 26.7]	20.8	23.1 [21.8, 24.5]	25.1	24.5 [22.3, 26.8]
	45–59	32	29.3 [27.3, 31.1]	56.6	36.2 [32.5, 40.0]	21.6	29.1 [27.0, 31.2]	42.5	34.7 [31.7, 37.8]	26.4	29.1 [27.7, 30.5]	49.5	35.4, [33.0, 27.8]
	Median (IQR) [95th percentile]	36 (27, 48) [58]	35 (27, 47) [58]	47 (37, 55) [58]	38 (28, 49) [57]	31 (25, 42) [57]	35 (27, 47) [58]	42 (31, 51) [58]	38 (28, 49) [58]	33 (26, 45) [58]	35 (27, 47) [58]	44 (34, 54) [58]	38 (28, 49) [58]
**Region**	England	88	85.5 [83.7, 87.2]	91	86.9 [82.4, 90.4]	87.2	86.1 [84.4, 87.6]	89.9	86.7 [83.8, 89.1]	87.6	85.8 [84.6, 86.9]	90.5	86.8 [84.3, 89.0]
	Wales	4.4	5.4 [4.4, 6.7]	2.7	4.4 [2.2, 8.6]	4.1	4.6 [3.7, 5.7]	3.4	4.6 [3.1, 6.9]	4.3	5.0 [4.3, 5.8]	3.1	4.5 [3.1, 6.6]
	Scotland	7.6	9.1 [7.7, 10.6]	6.3	8.7 [6.1, 12.1]	8.7	9.4 [8.1, 10.7]	6.7	8.6 [6.8, 10.9]	8.2	9.2 [8.3, 10.2]	6.5	8.7 [7.0, 10.6]
**Social grade**	A Upper middle class/ B Middle class	24.8	22.2 [20.5, 24.1]	27.1	22.4 [19.2, 26.0]	23.8	21.8 [20.1, 23.6]	24.9	19.2 [16.9, 21.7]	24.3	22.0 [20.8, 23.3]	25.9	20.7 [18.7, 22.9]
	C1 Lower middle class/C2 Skilled working class	54.8	55.6 [53.4, 57.9]	48	52.5 [48.0, 56.9]	50	51.5 [49.3, 53.7]	53	55.0 [51.6, 58.5]	52.3	53.6 [52.0, 55.2]	50.5	53.8 [51.0, 56.6]
	D Working class/E Lower level of subsistence	20.3	22.1 [20.2, 24.2]	25	25.1 [21.3, 29.4]	26.2	26.7 [24.8, 28.7]	22.1	25.8 [22.7, 29.1]	23.4	24.4 [23.0, 25.8]	23.5	25.4 [22.9, 18.2]
**Ethnicity**	White ^1^	88.2	83.6 [81.6, 85.5]	92.1	86.3 [81.8, 89.7]	88.6	84.8 [82.9, 86.5]	91.3	85.3 [81.9, 88.1]	88.3	84.2 [82.8, 85.5]	91.7	85.8 [83.1, 88.1]
	Mixed/multiple ^2^	2.1	1.8 [1.3, 2.5]	1.5	1.9 [1.0, 3.6]	2.3	1.7 [1.3, 2.2]	2.1	2.0 [1.2, 3.2]	2.3	1.8 [1.5, 2.2]	1.8	2.0 [1.3, 2.9]
	Asian/Asian British ^3^	7.1	9.1 [7.8, 10.7]	5	8.0 [5.2, 12.1]	6.2	8.5 [7.2, 10.0]	4.7	8.3 [6.2, 11.0]	6.6	8.8 [7.8, 9.9]	4.8	8.1 [6.3, 10.4]
	Black/Black British ^4^	2.1	3.8 [2.8, 5.1]	1.3	2.9 [1.6, 5.2]	2.4	4.1 [3.1, 5.3]	1.5	3.6 [2.2, 6.1]	2.2	3.9 [3.2, 4.8]	1.4	3.2 [2.2, 4.8]
	Other	0.5	1.6 [0.9, 2.9]	0.2	0.9 [0.2, 3.8]	0.6	1.0 [0.6, 1.8]	0.4	0.8 [0.3, 2.1]	0.6	1.3 [0.9, 2.0]	0.3	0.9 [0.4, 2.1]
**Sexual identity**	Heterosexual/straight	86.6	96.1 [95.5, 96.6]	87.1	93.1 [89.4, 95.6]	88.6	96.3 [95.7, 96.8]	91.4	95.5 [94.0, 96.7]	87.3	95.8 [95.3, 96.2]	89.2	94.2 [92.3, 95.7]
	Gay/Lesbian	7.5	2.2 [1.9, 2.6]	8.8	4.8 [2.6, 8.6]	2	0.9 [0.6, 1.2]	2.4	1.6 [0.8, 3.1]	4.7	1.7 [1.4, 1.9]	5.6	3.2 [1.0, 5.1]
	Bisexual	4.9	1.0 [0.8, 1.3]	3.9	1.8 [1.0, 3.2]	8.1	2.0 [1.7, 2.4]	5.1	2.3 [1.7, 3.3]	6.7	1.7 [1.4, 1.9]	4.5	2.1[1.5, 2.8]
	Other	1	0.7 [0.4, 1.2]	0.2	0.3 [0.1, 1.4]	1.3	0.8 [0.5, 1.3]	1.1	0.5 [0.3, 1.0]	1.3	0.9 [0.7, 1.2]	0.8	0.6 [0.3, 1.1]
**Married**	Yes	35.8	36.4 [34.3, 38.6]	44.5	41.3 [37.1, 45.7]	31.6	37.2 [35.0, 39.4]	45.7	42.7 [39.3, 46.1]	33.5	36.7 [35.1, 38.2]	45.1	42.0 [39.3, 44.7]
**Education ^5^**	No qualification	5	5.3 [4.3, 6.5]	3.3	3.8 [2.5, 5.7]	3.9	4.1 [3.3, 5.1]	3.1	3.6 [2.5, 5.3]	4.4	4.7 [4.0, 5.4]	3.2	3.7 [2.8, 4.9]
	Below degree	49.9	49.9 [47.6, 52.2]	50.1	50.8 [46.4, 55.2]	46.6	46.7 [44.6, 48.9]	44.9	44.7 [41.2, 48.2]	48.2	48.4 [46.8, 50.0]	47.6	47.8 [45.0, 50.6]
	Degree	45.1	44.8 [42.6, 47.1]	46.6	45.4 [41.1, 49.8]	49.5	49.2 [47.0, 51.4]	51.9	51.7 [48.2, 55.2]	47.4	46.9 [45.4, 48.5]	49.2	48.5 [45.7, 51.3]
**Rurality**	Urban	87.3	87.8 [86.2, 89.3]	84.8	86.6 [83.6, 89.1]	85.5	85.4 [83.6, 86.9]	81	81.6 [78.7, 84.1]	86.4	86.7 [85.5, 87.8]	82.9	84.1 [82.1, 86.0]
	Rural	12.7	12.2 [10.7, 13.8]	15.2	13.4 [10.9, 16.4]	14.5	14.6 [13.1, 16.4]	19	18.4 [15.9, 21.3]	13.6	13.3 [12.2, 14.5]	17.1	15.9 [14.0, 17.9]
**General health ** **status**	Good/ Very Good	5.3	5.1 [4.2, 6.1]	7.5	5.6 [4.2, 7.3]	4.8	5.0 [4.1, 6.0]	5.7	5.2 [3.9, 6.7]	5.2	5.1 [4.5, 5.9]	6.6	5.5 [4.5, 6.6]
	Fair	20.5	19.8 [18.0, 21.6]	21.8	18.2 [15.2, 21.6]	21.2	21.5 [19.8, 23.4]	23.2	22.9 [20.1, 25.9]	20.8	20.6 [19.4, 21.9]	22.5	20.5 [18.4, 22.8]
	Bad/ Very bad	74.2	75.1 [73.1, 77.0]	70.7	76.3 [72.6, 79.6]	74	73.5 [71.5, 75.4]	71.1	72.0 [68.8, 75.0]	74	74.2 [72.8, 75.6]	70.9	74.0 [71.6, 76.3]
**Limiting long-** **term illness/** **disability**	Yes	29.6	27.6 [25.6, 29.7]	34.1	27.6 [24.2, 31.4]	36.9	35.6 [33.5, 37.8]	37.4	34.7 [31.5, 38.0]	33.5	31.7 [30.3, 33.2]	35.8	31.3 [28.9, 33.8]
**Ever had any ** **partnered ** **sexual ** **experience (not ** **necessarily ** **involving ** **genital ** **contact) ^6^**	Yes	89.9	89.3 [87.8, 90.7]	93.2	90.6 [87.2, 93.1]	89	88.2 [86.6, 89.6]	92.2	90.9 [88.4, 92.9]	89.3	88.7 [87.6, 89.7]	92.6	90.6 [88.6, 92.3]
**Ever had ** **vaginal, anal, ** **oral sex or ** **other genital ** **contact with a ** **partner of any ** **gender) ^6^**	Yes	83.8	82.7 [80.7, 84.6]	89.8	87.2 [83.5, 90.2]	81.5	80.8 [78.8, 82.7]	86.5	83.9 [80.6, 86.7]	82.5	81.7 [80.3, 83.0]	88.1	85.4 [83.0, 87.5]
**Ever had ** **vaginal, anal, ** **oral sex or ** **other genital ** **contact with ** **a same sex ** **partner ^6^**	Yes	13.3	6.6 [5.6, 7.6]	12.9	9.2 [6.2, 13.4]	9.8	5.7 [4.9, 6.7]	7.7	4.8 [3.6, 6.3]	11.4	6.1 [5.5, 6.8]	10.3	6.9 [5.2, 9.1]
**Partner ** **numbers, ** **lifetime ^7^**	1 partner	13	13.3 [11.6, 15.2]	12.3	16.7 [12.6, 21.9]	17.1	17.8 [15.9, 20.0]	16.4	17.1 [14.3, 20.2]	15.1	15.4 [14.1, 16.9]	14.3	16.9 [14.3, 19.8]
	2 partners	10.9	11.8 [10.1, 13.6]	7.7	10.5 [7.8, 14.1]	13	12.2 [10.6, 14.0]	12.6	12.1 [9.8, 14.9]	12	12.0 [10.8, 13.2]	10.2	11.3 [9.4, 13.5]
	3–4 partners	14.5	15.7 [13.8, 17.9]	14	14.9 [12.0, 18.4]	18.5	19.3 [17.3, 21.4]	15	16.3 [13.5, 19.4]	16.6	17.5 [16.0, 18.9]	14.5	15.6 [13.5, 17.9]
	5+ partners	61.6	59.2 [56.5, 61.9]	66	57.8 [52.8, 62.7]	51.4	50.7 [48.1, 53.3]	56	54.6 [50.7, 58.5]	56.3	55.1 [53.3, 57.0]	61.1	56.2 [53.0, 59.3]
	Median (IQR) [95th percentile]	7 (3, 16) [100]	6 (3, 15) [60]	7 (3, 15) [66]	6 (2, 12) [50]	5 (2, 10) [31]	5 (2, 10) [30]	5 (2, 10) [30]	5 (2, 10) [25]	5 (2, 12) [50]	5 (2, 12) [45]	6 (3, 13) [50]	5 (2, 11) [35]
**Ever had ** **vaginal, anal, ** **oral sex or ** **other genital ** **contact with ** **partner of any ** **gender, July ** **2019-July 2020 ^6^**	Yes	71.6	70.6 [68.3, 72.9]	69.1	69.3 [65.0, 73.3]	71.6	69.5 [67.2, 71.7]	67.8	68.1 [64.5, 71.5]	71.6	70.0 [68.4, 71.6]	68.3	68.6 [65.8, 71.2]
**Partner ** **numbers, July ** **2019-July 2020 ^8^**	1 partner	74.6	77.3 [74.7, 79.7]	85.7	86.2 [81.9, 89.6]	85.1	88.0 [86.2, 89.6]	92.7	91.1 [87.6, 93.8]	80	82.3 [80.7, 83.8]	89.1	88.7 [86.0, 90.9]
	2 partners	11.9	11.8 [10.0, 13.8]	7	6.6 [4.6, 9.5]	7.5	6.5 [5.3, 7.9]	4.4	6.5 [4.2, 10.1]	9.7	9.3 [8.2, 10.6]	5.7	6.6 [4.9, 8.7]
	3–4 partners	5.8	5.0 [3.8, 6.5]	2.8	3.3 [1.7, 6.4]	4.4	3.3 [2.5, 4.4]	1.3	1.1 [0.5, 2.5]	5	4.2 [3.5, 5.1]	2.1	2.2 [1.3, 3.8]
	5+ partners	7.7	6.0 [4.7, 7.4]	4.5	3.9 [2.1, 7.2]	3	2.2 [1.6, 3.1]	1.6	1.2 [0.6, 2.4]	5.3	4.2 [3.5, 5.0]	3.1	2.6 [1.5, 4.2]
	Median (IQR) [95th percentile]	1 (1, 2) [10]	1 (1, 1) [9]	1 (1, 1) [5]	1 (1, 1) [5]	1 (1, 1) [4]	1 (1,1) [3]	1 (1, 1) [2]	1 (1, 1) [2]	1 (1, 1) [6]	1 (1, 1) [5]	1 (1, 1) [3]	1 (1, 1) [3]
**1 or more new ** **partner(s), July ** **2019-July 2020 ^8^**	Yes	36.4	35.2 [32.4, 38.1]	22.8	30.1 [24.6, 36.3]	25.7	22.2 [20.0, 24.5]	15.4	17.4 [14.0, 21.4]	30.8	28.9 [27.1, 30.8]	19.2	23.8 [20.4, 27.5]

Legend: CI=confidence intervals. 1. White includes all those who identify as White English, Welsh, Scottish, Northern Irish, British, Irish, Gypsy or Irish Traveller, or from any other White background. 2. Mixed ethnicity includes those who identify as White and Black African, White and Black Caribbean, White and Asian, or any other mixed or multiple ethnic background. 3. Asian includes those who identify as Indian, Pakistani, Bangladeshi, Chinese or from any other Asian background. 4. Black includes those who identify as African, Caribbean, or from any other Black background. 5. Natsal-COVID participants chose an option from the following list: (1) primary school, (2) secondary school (age under 15), (3) GNVQ / GSVQ / GCSE/ SCE standard, (4) NVQ1/NVQ2, (5) NVQ3/ SCE Higher Grade/ Advanced GNVQ/ GCE A/AS or similar, (6) NVQ4 / HNC / HND / Bachelor's degree or similar, and (7) NVQ5 or post-graduate diploma. A 3-category education variable, based on a variable used by 2018 Health Survey for England (HSE), including “no qualification,” “below degree”, and “degree” was derived using the following: No qualification: 1–2; Below degree: 3–5; Degree: 6–7. 6. All respondents. 7. All respondents reporting at least one sexual partner in their lifetime (vaginal, anal, oral sex, or other genital contact). 8. All respondents reporting at least one sexual partner in the past year (vaginal, anal, oral sex, or other genital contact).

## Discussion

Natsal-COVID is a large, multi-wave, national study that was undertaken when data were urgently needed to understand the impact of the pandemic on SRH services and inform policy. Initial findings from Natsal-COVID Wave 1 have been used in SRH policy and practice in Britain ^14, 15^. Natsal-COVID Wave 2 data generated one-year prevalence estimates for a range of key SRH behaviours and outcomes one year after the start of the first COVID-19 lockdown in Britain. The second wave of the survey also provides an opportunity to examine change over time during the first year of the pandemic.

Key sociodemographic characteristics and reported sexual behaviours were generally similar in the weighted Natsal-COVID data when compared to external probability surveys and Natsal-3. However, we noted bias in several important characteristics that remained after weighting. The Natsal-COVID Wave 2 sample under-represented individuals who were married and those who self-reported ‘very good’ or ‘good’ general health. Among those with at least one sexual partner in their lifetime, a higher proportion of Natsal-COVID Wave 2 participants reported having no sexual partners in the past five years compared with Natsal-3 (conducted ten years ago). This difference could reflect pandemic restrictions, differences in the question wording, mode effects ( *i.e.*, online versus in-person), sampling bias or, most likely, a combination of these factors.

Prior to weighting, like Wave 1, Natsal-COVID Wave 2 had a higher proportion of participants identifying as non-heterosexual compared to 2019 APS. The over-representation of people with non-heterosexual identities in the unweighted Natsal-COVID data is consistent with previous web-panel surveys ^16, 17^. However, weighted percentages of non-heterosexual individuals were comparable between Natsal-COVID Wave 2 and 2019 APS.

The profile of participants who took part in both waves of Natsal-COVID (the longitudinal sample) demonstrated high survey attrition. Sample bias could be due to differential attrition, particularly among younger adults. Among sexually active participants, the longitudinal sample were less likely to report multiple sexual partners in the past year or a new sexual partner in the past year compared to participants who only participated in Wave 1 of Natsal-COVID. These differences in sexual behaviour estimates are likely attributable to sample bias predominately driven by age. Researchers conducting longitudinal analysis using Natsal-COVID data should bear in mind sample attrition and its associated bias when interpreting findings. The application of weights to the longitudinal sample reduced the magnitude of difference but did not correct them entirely.

Natsal-COVID did not use probability sampling methods and therefore inference to the general population should be undertaken with caution ^18^. There are known sources of bias in web-panel surveys that may affect survey estimates ^17, 19^. Although the Natsal-COVID findings are likely to be largely generalisable, caution in the interpretation of prevalence estimates is advised particularly when analysing the longitudinal sample. Additionally, the population surveys which we have used as benchmark estimates are subject to measurement and representation errors. Natsal-3 was also conducted more than ten years ago, so differences in the sexual behaviour estimates may also be attributable to temporal changes.

In conclusion, the Natsal-COVID Wave 2 survey has enabled us to identify impacts of the COVID-19 pandemic throughout the year following the first national lockdown in Britain and to monitor change over time in the same period.

## Data Availability

The Natsal-COVID Wave 1 dataset has been deposited with the UK Data Archive (an open access repository) with safeguarded access (SN 8865 - National Survey of Sexual Attitudes and Lifestyles COVID-19 Study, 2020). The dataset is available to users registered with the UK Data Service. http://doi.org/10.5255/UKDA-SN-8865-2 ^7^. Wave 2 data will be deposited at the same location within 10–12 weeks. Given the sensitive nature of the content of the data, a thorough disclosure risk assessment and the application of disclosure control measures are necessary prior to safe deposit. In the meantime, interested researchers or reviewers may contact the Natsal team ( natsal@ucl.ac.uk) for interim access, with appropriate considerations about confidentiality and data protection. Other datasets (2019 Annual Population Survey, 2018 Health Survey for England and 2010–12 Natsal-3 study) used in this analysis are publicly available via the UK Data Archive. Datasets can be accessed through registration with the UK Data Service UK Data Service: Annual Population Survey, January–December, 2019. http://doi.org/10.5255/UKDA-SN-8632-5 ^10^. UK Data Service: Health Survey for England, 2018. http://doi.org/10.5255/UKDA-SN-8649-2 ^11^. UK Data Service: National Survey of Sexual Attitudes and Lifestyles, 2010-2012. http://doi.org/10.5255/UKDA-SN-7799-2 ^13^. The datasets held in the UK Data Service are under safeguarded access and can be accessed by accepting the End User Licence. The published 2019 Annual Population Survey sexual identity tables are available as a downloadable Excel file on the ONS website ^12^. This dataset is available under the terms of the Open Government License v3.0.
