# Supplementary material for: Methodology of Natsal-COVID Wave 2: A large, quasi-representative, longitudinal survey measuring the impact of COVID-19 on sexual and reproductive health in Britain
Source: Wellcome Open Res. 2024 Jan 8;7:166. Originally published 2022 Jun 1. [Version 2] doi: 10.12688/wellcomeopenres.17850.2 (PMC10545983; doi:10.12688/wellcomeopenres.17850.2)
Supplement: Peer review history

# Reviewer report: Reviewer response for version 1

**DOI:** 10.21956/wellcomeopenres.19765.r65798

**Published:** 2023-10-04

**Authors:** Pablo Cabrera Álvarez

**Affiliations:** 1 Institute for Social and Economic Research, University of Essex, Colchester, England, UK

This research note provides a comprehensive overview of the methodology of the wave 2 of the Natsal-COVID study. The primary objective of this second wave was to capture one-year prevalence estimates for a range of sexual and reproductive health measures. The survey, which took place in March-April 2021, involved both a re-issue of the wave 1 questionnaire to respondents willing to be recontacted and the recruitment of a fresh sample from a web panel. In addition to detailing the methodology, this paper includes a representativeness analysis using a diverse set of measures from high-quality probability samples. Additionally, the authors address the principal limitations of the research design and their potential impact on data analysis, building upon the explanations provided in the research note on wave 1 recruitment (Dema et al., 2022).

 The paper provides a detailed and clear overview of the wave 2 design, which will be helpful for researchers and other data users. Now, I discuss four minor points that might help the authors to refine the paper:

- **Brief description of the panel recruitment methodology**. The recruitment methodology of the panel is likely to affect the quality of survey estimates (see Mercer and Lau, 2023; Cornesse et al., 2020). It is understood that a comprehensive explanation of the panel recruitment methodology may be beyond the scope of this research note, but it would be informative to have a few more details about the panel recruitment methodology (if these details are available to the authors).
- **The benchmark estimates are also subject to error**. The benchmark estimates used in the representativeness analysis come from probability surveys and are also subjected to measurement and representation errors. Furthermore, when considering Natsal-3 (2010-12), in addition to survey errors, it should be factored in the influence of time and its potential effects on the target population. This temporal aspect may partially account for some of the observed differences.
- **Confidence intervals (CIs) in the representativeness analysis**. In the representativeness analysis, where comparisons are made between Natsal-COVID survey estimates and benchmark data (Tables 1 and 2), the 95% confidence intervals are provided for the estimates from the probability surveys and the Natsal-COVID survey. The authors could provide some information about how they estimated the standard errors, especially in the case of the estimates from the Natsal-COVID study, a nonprobability sample (see Wu, 2022). Providing some insight into the estimation of confidence intervals would help readers better interpret the information presented in the table.
- **Quota sampling and panel attrition**. In the discussion section (page 13, paragraph 3 of the PDF), the authors suggest that attrition between waves 1 and 2 may be attributed to quotas not being applied in the recruitment of the longitudinal sample. However, the paper that presents the design of wave 1 states that quotas were used in recruitment (Dema et al., 2022), and it is not clear how the quotas could be implemented in the second wave of a longitudinal study since they would harm the sample size and the feasibility of conducting longitudinal analyses. An explanation of this point could help clarify their idea.

Is the work clearly and accurately presented and does it cite the current literature?

Yes

If applicable, is the statistical analysis and its interpretation appropriate?

Partly

Are all the source data underlying the results available to ensure full reproducibility?

Yes

Is the study design appropriate and is the work technically sound?

Yes

Are the conclusions drawn adequately supported by the results?

Yes

Are sufficient details of methods and analysis provided to allow replication by others?

Yes

Reviewer Expertise:

Survey methodology.

I confirm that I have read this submission and believe that I have an appropriate level of expertise to confirm that it is of an acceptable scientific standard.

## References

[1] : Methodology of Natsal-COVID Wave 1: a large, quasi-representative survey with qualitative follow-up measuring the impact of COVID-19 on sexual and reproductive health in Britain. *Wellcome Open Res* .2021;6: 10.12688/wellcomeopenres.16963.2 209 10.12688/wellcomeopenres.16963.2 35495088 PMC9020538

[2] : A Review of Conceptual Approaches and Empirical Evidence on Probability and Nonprobability Sample Survey Research. *Journal of Survey Statistics and Methodology* .2020;8(1) : 10.1093/jssam/smz041 4-36 10.1093/jssam/smz041

[3] : Statistical inference with non-probability survey samples. *Survey Methodology* .2022;2(48) : Reference source

[4] : Comparing two types of online survey samples. *Pew Research Center* .2023; Reference source

# Reviewer report: Reviewer response for version 1

**DOI:** 10.21956/wellcomeopenres.19765.r67364

**Published:** 2023-10-02

**Authors:** Sylvester Reuben Okeke

**Affiliations:** 1 College of Health Sciences, ECA College of Health Sciences, Sydney, NSW, Australia; 2 Centre for Social Research in Health, UNSW, Sydney, Australia

This very interesting and relevant methodology paper reports a second wave of data on prevalence of key SRH outcomes. The paper also compares the second wave data with the first wave data to track changes in SRH outcomes over a one-year period.

 The methodological strength of this manuscript is its rigour, which is evident in use of a representative sample, methods of data collection and analytical techniques. Notably, authors identified and reported known sources of bias and caveats within which the research results and conclusions are to be understood and interpreted.

Is the work clearly and accurately presented and does it cite the current literature?

Yes

If applicable, is the statistical analysis and its interpretation appropriate?

Yes

Are all the source data underlying the results available to ensure full reproducibility?

Yes

Is the study design appropriate and is the work technically sound?

Yes

Are the conclusions drawn adequately supported by the results?

Yes

Are sufficient details of methods and analysis provided to allow replication by others?

Yes

Reviewer Expertise:

Global Public Health, Sexual and Reproductive Health, Population Health, Blood-borne Viruses and Sexually Transmissible Infections, Adolescents Sexual and Reproductive Health and Rights, Research Methods.

I confirm that I have read this submission and believe that I have an appropriate level of expertise to confirm that it is of an acceptable scientific standard.

# Reviewer report: Reviewer response for version 1

**DOI:** 10.21956/wellcomeopenres.19765.r54470

**Published:** 2023-02-22

**Authors:** Holly Hope

**Affiliations:** 1 Centre for Women’s Mental Health, School of Health Sciences, University of Manchester, Manchester, UK

The Natsal-COVID Wave 2 methodology paper clearly describes the design and procedures of data collection and compares the wave 2 ands wave 1 cohort characteristics. The limitations of the methods are detailed in the discussion so that researchers using the dataset for longitudinal versus cross sectional analyses are aware of limits to generalisability. Where information is not presented in this publication it is signposted in the references.

 This is an important study for a number of reasons; we now know that the UK live birth rate temporarily dropped during pandemic; to what extent that reflected changes in sexual and reproductive health practices is an important question that this dataset could address. The reporting by sexual and gender identity reflects the society we live in and ideally all contemporary surveys should be designed to report on the experience and views of LGTBQ+ people.

 There are few contemporary longitudinal datasets that capture information on IPV, making this dataset very useful.

 I hope that further waves of data are collected so that we can understand to what extent there are long term pandemic effects on intimate relationships and sexual health.

Is the work clearly and accurately presented and does it cite the current literature?

Yes

If applicable, is the statistical analysis and its interpretation appropriate?

Yes

Are all the source data underlying the results available to ensure full reproducibility?

Yes

Is the study design appropriate and is the work technically sound?

Yes

Are the conclusions drawn adequately supported by the results?

Yes

Are sufficient details of methods and analysis provided to allow replication by others?

Yes

Reviewer Expertise:

Psychiatric epidemiology, reproductive health, behavioural science and women's health.

I confirm that I have read this submission and believe that I have an appropriate level of expertise to confirm that it is of an acceptable scientific standard.
